# Gynecologic symptoms and the influence on reproductive life in 386 women with hypermobility type ehlers-danlos syndrome: a cohort study

**DOI:** 10.1186/s13023-016-0511-2

**Published:** 2016-09-13

**Authors:** Justine Hugon-Rodin, Géraldine Lebègue, Stéphanie Becourt, Claude Hamonet, Anne Gompel

**Affiliations:** 1 Unité de Gynécologie-Endocrinienne, APHP, Hôpitaux universitaires Paris Centre, Université Paris Descartes, Port Royal, 123 Bd de l’Hôpital, Paris, 75014 France; 2 Service de médecine physique et réadaptation, APHP, Hôpitaux Universitaires Paris Centre, Université Paris-Est Créteil, Hôtel Dieu, Place Jean XXIII, Paris, 75004 France

**Keywords:** Ehlers-Danlos syndrome, Hypermobility type, Recurrent abortion, Endometriosis, Bleeding disorders, Premature delivery

## Abstract

**Background:**

Hypermobile Ehlers-Danlos syndrome (hEDS), is probably the most common disease among heritable connective tissue disorders. It affects women more than men and causes symptoms in multiple organs. It is associated with chronic pain, skin fragility and abnormal bleeding. These characteristics may hamper reproductive life. We conducted a study to evaluate the gynecologic and obstetric outcomes in women with hEDS. We also explored a possible hormonal modulation of the hEDS symptoms. The gynecologic and obstetric history of 386 consecutive women diagnosed with hEDS was collected by a standardized questionnaire and a medical consultation performed by a senior gynecologist in an expert centre for hEDS between May 2012 and December 2014.

**Results:**

We observed a high frequency of gynecologic complaints, specifically: menorrhagia (76 %), dysmenorrhea (72 %) and dyspareunia (43 %). Endometriosis was not highly prevalent in this population. The obstetric outcomes were similar to those of the general French population for deliveries by cesarean section (14.6 %) and premature births (6.2 %) but the incidence of multiple spontaneous abortion (13 %) and spontaneous abortion (28 %) were significantly higher. A subset of women were sensitive to hormonal fluctuations with more severe symptoms occurring during puberty, prior to menstruation, during the postpartum period as well as on oral contraception.

**Conclusions:**

Increased awareness of the gynecological symptomatology in women with hEDS can help discriminate between endometriosis and thus prevent useless, and potentially dangerous, surgery. This study also suggests that hormonal modulation may be an appropriate treatment for a subset of women with hEDS.

## Background

Ehlers-Danlos syndromes (EDS) belong to heritable connective tissue disorders. Geneticists (Villefranche 1997) have categorized six major forms of EDS including the classic type (I, II), hypermobility type (hEDS) (III) and vascular type (IV) as being the most frequent clinical presentations [1]. However, while the vascular or classical types of EDS may be associated with genetic variations, the diagnosis of hEDS is based solely on clinical criteria. hEDS is classically defined on the basis of major criteria: a Beighton score of ≥5/9, skin involvement (hyperextensibility and/or smooth, velvety skin) and minor criteria: recurring joint dislocations, chronic joint/limb pain and a positive family history [1]. According to an international group of experts, and because of overlapping symptoms, Join hypermobile syndrome (JHS) and hEDS could be the same clinical entity [2]. Recent studies suggest that patients with hEDS can experience a large range of dysfunction and in addition to mobile, loose joints, chronic pain (musculoskeletal pain, headache/migraine) [3] and variable skin involvement, also including dysautonomia, gastrointestinal functional disorder, fibromyalgia, and chronic fatigue syndrome among others [4]. Furthermore, not only does the clinical spectrum of the disease and its functional consequences vary widely but there is also a wide inter- and intra-familial variability in the severity of symptoms [5–8].

Its prevalence is classically estimated at 1 in 5000 [1] but may be higher according to recent debate about the overlap with JHS reaching 0.75–2 % [7]. hEDS affects women more often than men [6,8].

Previous studies have found an increase in abnormal bleeding, dyspareunia and dysmenorrhea in patients with EDS [9–12]. Four studies have reported on the gynaecologic and obstetric outcomes in women with both JHS, hEDS and other EDS types with some discrepancies on the prevalence of gynecologic complaint, spontaneous abortion and obstetrical outcomes [9–14]. This can be due to pooling different types of EDS in the analysis and getting the information through a self-declaring questionnaire. Indeed the last largest study published was based on an email questionnaire with no clinical validation [14].

We were therefore interested in conducting a study to evaluate the prevalence of gynecologic disorders and the impact on obstetric outcome using a standardized questionnaire during a medical consultation taking advantage of a large cohort of women with hEDS. Because of controversial reports on the role of estrogen and progestins in hyperlaxity we also thought it would be interesting to explore whether hormonal factors play a role in the fluctuating symptoms of hEDS [15] .

## Methods

The aim of this cohort study was to describe the gynecologic symptoms and the obstetric history of a series of women with hEDS. We also explored whether hEDS symptoms were sensitive to the hormonal environment.

1. Patients
  hEDS is a clinical diagnosis with no confirmatory test available. Hence, all women had been clinically assessed at least once by a single national expert in hEDS between May 2012 and December 2014 then hEDS women were sent to a consultation in Gynecology and included. All had been formally diagnosed as having hEDS as previously published [16–18]. The diagnosis was based on actual or previous hypermobility, joint instability, chronic pain, skin abnormalities and several other criteria as chronic fatigue, proprioceptive disorders, dysautonomia, migraines, digestive disorders. Almost all patients had a family history of hEDS with variability in severity of clinical symptoms among relatives.
2. Methods
  A standardized original questionnaire was completed during the patient consultation.
  The questionnaire included questions about:
  hEDS symptoms, luxation, joint pain, fatigue, headache/migraine and digestive disorders.
  Gynecologic symptomatology, throughout the reproductive life and at the time the questionnaire was administered, including dysmenorrhea, metrorrhagia, menorrhagia (menstruation > 7 days, or > 5 sanitary napkins/day) and dyspareunia.
  Gestation and parity, pregnancy outcomes and deliveries.
  The influence of hormones was evaluated by asking the women about changes in the severity of their predominant hEDS symptoms (chronic pain and fatigue) (worsening, no effect, or improvement) during puberty, while taking hormonal contraceptives, during pregnancy, the postpartum period, and menopause.
  A gynecologic examination was performed for each patient by a senior gynecologist (A.G). In cases of a clinical suspicion of endometriosis (dysmenorrhea, deep dyspareunia, or pelvic nodules found during physical examination) or severe menorrhagia/dyspareunia, a pelvic sonogram and, if necessary, magnetic resonance imaging (MRI) were performed by an experienced radiologist.
  In our cohort, hemostatic disorders were systematically ruled out in women presenting with menorrhagia or hemorrhagic disorders.
3. Statistical analyses
  Statistical analysis used procedures available in SAS software (SAS Institute, Inc., Cary, NC). The student *t*-test and the *χ*^2^ test were used to assess the differences in characteristics of women between two hEDS groups (not influenced by menstruation and influenced by menstruation). Data are given as percentage, mean and standard deviation (SD).
  A *p* value of 0.05 was taken to represent statistical significance.

## Results

Overall, 386 women were included for analysis. The general characteristics of the population are described in Table 1.

**Table 1:** General characteristics of the hEDS population (*n* = 386)

Age at inclusion [mean (SD); in years]	37.8 (14.1)
Age at diagnosis [mean (SD); in years]	35.3 (14.2)
Age at first symptoms [mean (SD); in years]	12.5 (11.8)
Age at puberty [mean (SD); in years]	12.5 (1.7)
hEDS symptoms	
Joint pain (%)	370 (96)
Fatigue (%)	353 (92)
Luxation (%)	333 (86)
Digestive disorders (%)	345 (90.1)
Migraine and headache (%)	289 (75.5)

The hEDS symptoms were predominantly luxation in 86 % of women, joint pain in 96 %, fatigue in 92 % and digestive disorders in 90 %.

Bleeding disorders were the most prominent gynecologic symptoms (Table 2). Menorrhagia was reported by 76 % of the women with an incidence which did not vary according to age in premenopausal women (*p* = 0.9).

**Table 2:** Gynecological symptoms and prevalence of endometriosis

Symptoms	*n* (%)
Menorrhagia	292 (76)
Metrorrhagia	83 (22)
Dysmenorrhea	278 (72)
Deep dyspareuniea	118 (38)
Intromission dyspareuniea	148 (43)
Endometriosis	20 (6)

Dysmenorrhea was present in 72.8 % of the women (Table 2). The frequency of dysmenorrhea did not vary significantly with age (81 % of the women under 20 years, 74 % of those between 30 and 40 years and 67 % of those above 40 years; *p* = 0.1). Among the 369 women who had experienced sexual intercourse, severe dyspareunia was reported by 61 %.

Endometriosis was diagnosed in 6 % of the women. This percentage includes patients with a history of surgical procedures for suspicion of endometriosis and those for whom a clinical examination and/or a pelvic sonogram/MRI was performed.

### Obstetric outcomes

In the study population, there were 747 pregnancies for 225 women. The average number of pregnancies per woman was 2.3 ± 2.3, with the average number of deliveries being 1.4 ± 1.4. Of these, 441 deliveries were achieved in 196 women. Spontaneous abortions were frequent, occurring in 28 % of pregnancies. Forty-five percent of the women had experienced at least one abortion; recurrent abortion (defined as ≥3 abortions with the same partner) occurred in 13 % of the women. Recurrent abortions were not consecutive, with live births occurring in most of the women (87 %). Eighty-five percent of the recurrent abortions occurred before the age of 40. The mean age for the last abortion was 25.9+/−5.9 years.

The majority of the pregnancies followed a favourable course with severe complications occurring in a limited number of cases. Twenty-six of the deliveries (6.2 %) were preterm (i.e., before 37 weeks of amenorrhea). Despite increased bleeding symptoms in these patients, only 4.8 % of the births were complicated with postpartum haemorrhage. Similarly, despite skin fragility, only 2.4 % of deliveries were complicated with severe vaginal tears. Cesarean section was performed in 14.6 % of births.

### Influence of reproductive life on hEDS

Out of 70.4 % of the women who had experienced symptoms of hEDS before the onset of puberty, 52 % associated puberty with a worsening of the symptoms. The onset of hEDS symptoms coincided with puberty in 16.9 % of the cohort. Overall then, 51.5 % of the population reported that puberty had a deleterious influence: those for whom puberty was associated with a worsening of the symptoms and those for whom puberty marked the onset of symptoms.

In the whole cohort, 162 women (42 %) had used combined hormonal contraceptives (CHC), for an average duration of 8.8 ± 7.6 years. Progestin-only contraceptives (POP) in the form of a low-dose mini pill or antigonadotropic agents at higher doses had been prescribed for medical indications (e.g., menorrhagia, dysmenorrhea, benign uterine conditions or due to a contraindication to CHC) in 67 women (17 %). An improvement in hEDS symptoms was reported by 13.6 % of the women using CHC and 25.4 % of those using POP, (*p* = 0.03) (OR, 0.46 [CI 95 %, 0.23–0.94]).

hEDS symptoms worsened for 26 % of the women during pregnancy and for 37.6 % during the postpartum period.

Overall, 16.8 % of the women included in the study were postmenopausal. Among these, 22 % reported an improvement in their symptoms after menopause. Menopausal hormone therapy (MHT) had been used by 41.5 % of these patients for a mean duration of 6.8 ± 5.5 years. Four of these patients observed an improvement in their hEDS symptoms as a result of MHT.

### Sub-analysis of the women who experienced cyclic effect on chronic pain and fatigue

Over one-third of the women (*n* = 133) experienced a worsening of symptoms during each perimenstrual period.

We carried out a sub-analysis of these women, to determine whether they were also affected by puberty, CHC or pregnancy compared to those women who reported no cyclic influence (*n* = 197). There was no difference among the two groups in terms of age at diagnosis, age at onset of symptoms, number of pregnancies or CHC duration of use.

Seventy-eight (59 %) of the women reporting cyclic variations (*p* = 0.01) (Table 3) reported that their symptoms began or worsened at puberty. CHC use was associated with a significant worsening of symptoms among patients with menstrual aggravation. One-fourth of the women whose symptoms were influenced by menstruation stated that their symptoms were modified when taking CHC, in contrast with only 5.6 % in the group whose hEDS symptoms were uninfluenced (*p* = 0.001) (Table 3). Furthermore, women experiencing cyclic modulations also experienced worsened symptoms during the postpartum period (*p* = 0.05).

**Table 3:** The influence of hormones on hEDS symptoms (hEDS symptoms: chronic pain, fatigue)

	hEDS patients not influenced by menstruation *n* = 197 (%)	hEDS patients influenced by menstruation *n* = 133 (%)	*p*
Influenced by puberty	85/197 (43.2)	79/133 (58.7)	0.01
Impact of CHC			
Worsened on CHC	5/90 (5.6)	15/58 (25.9)	0.001
Improved on CHC	12/90 (13.3)	9/58 (15.5)	
Unchanged on CHC	73/90 (81.1)	34/58 (58.6)	
Improved by menopause	6/33 (18.2)	3/17 (17.7)	NS

## Discussion

This study describing the gynaecologic and obstetric symptoms in a large cohort of women with hEDS suggests that most women experience significant gynaecologic symptoms and that few severe complications occur during pregnancy. In addition, this is the first study to describe the impact of reproductive life on hEDS clinical outcomes.

Abnormal bleeding, dysmenorrhea and dyspareunia were the most common gynecologic complaints in our hEDS population. Dysmenorrhea was not correlated with age and did not improve after deliveries contrary to what is observed with idiopathic dysmenorrhea [19]. A recent comprehensive literature review reported that severe dysmenorrhea affected between 2 and 29 % of women and that dysmenorrhea was negatively associated with women’s age and parity [20]. Easy bruising and bleeding are frequently described in EDS, a result of weakness in the capillaries and perivascular connective tissue rather than from hemostatic dysfunction [11]. The sexual life of these women is also adversely affected by a high incidence of dyspareunia. Previous studies have also found an increase in abnormal bleeding, dyspareunia and dysmenorrhea in patients with EDS [9, 10, 13, 14]. The two last publications have selectively reported on the gynaecologic and obstetric outcomes in patients with hEDS/JHS. The first involved 82 women in Italy with 93 pregnancies [10] and the second study included about 770 women with both hypermobility type and other EDS types as well based on an email questionnaire with no clinical validation [9]. Both studies reported similar prevalence of dysmenorrhea and dyspareunia in women with hEDS/JHS than in our population. The combination of these symptoms is highly suggestive of endometriosis. The rate of endometriosis we found was the same as that for the general population (3–6 %) and much less than in women with chronic pain [21]. While it is possible that we underestimated the frequency of endometriosis because we did not perform systematic laparoscopy, we suggest that endometriosis may be over-diagnosed in hEDS patients because of reports of chronic pain and bleeding. A systematic study would nevertheless be interesting to evaluate the prevalence of adenomyosis at a young age in women with EDS as it may play a role in the high risk of spontaneous abortion. A previous study, which also used a questionnaire, found a 15 % prevalence of reported endometriosis [9]. A 22 % prevalence of endometriosis among women with suspected infertility was reported by women with EDS from an emailed questionnaire which did not allow to validate the diagnosis of endometriosis. In our patient population, the conception rate was close to that for women in France in general (fertility rate: 1.8–2.03 between 1980 and 2013 [22]). This finding further argues against the hypothesis of an abnormally high prevalence of endometriosis in this population. However, while the rate of conception in our population was similar to the normal range, the rate of spontaneous abortions was higher than in the general population (28 % versus about 20 %, respectively). Furthermore, the rate of multiple abortions was much higher in our population than in the French population as a whole (13 % versus about 1 %, respectively) [23]. The cause of the miscarriages is unclear. An increased contractility of the uterus or a fragile cervix, related to the connective tissue defect and dysautonomic syndrome could be a cause [24]. Another possible explanation could be implantation defects.

One novel aspect of our study is the relationship between hEDS symptoms and reproductive life. It is significant that estradiol receptors are present in many of the body structures and organs including joints, skin, and cartilage. Puberty does appear to significantly exacerbate symptoms. This may result either from the rapid growth that is characteristic of this time in life — and that significantly affects skin, joints and muscles—and/or to the rapid increase in estrogen secretion. In the subset of patients who deteriorate during the perimenstrual period, CHC was also correlated with an increase in symptoms. Our analysis of contraception suggests that, in some women at least, the hEDS symptoms responsible for increased disability might improve with the use of POP. Our findings suggest that, when menstrual disorders are treated and alleviated either by CHC or by POP, EDS symptoms improve and women report less fatigue. In the literature, there is conflicting data as to the effects of hormones on connective tissue, joint laxity and tendons. It has been demonstrated that estradiol decreases the formation of collagen in tendons following exercise [25]. Joint laxity increases during pregnancy in some women. Another smaller study found increased knee laxity during ovulation compared with the luteal phase, but no significant changes during the phases of the menstrual cycle [26]. A prospective trial would be useful to determine the precise nature of the role of estrogens and progestins on various symptoms of hEDS.

This study also reveals that the prevalence of obstetric complications is not substantially greater compared to the healthy population and lower [27] in comparison with the previously mentioned large, recently-published study [14]. This discrepancy may result from the different methodologies used in the two studies, or from a difference in the protocols for management of pregnancies from one country to another. Indeed, the Italian study fitted more with our results with 10.7 % preterm deliveries; we agree with these authors than caesarean section is not indicated systematically in women with hEDs and was performed only in 14 % of our patients.

### Strengths and weaknesses of this study

- Strengths
  The cohort used in this study is quite large for a rare disease. The standardized questionnaire was filled out face to face with the patient; and the medical reports were validated and corroborated by a medical examination performed by a senior gynaecologist trained in the diagnosis of endometriosis. This offers a stronger basis for validation of symptoms than the study carried out by Hurst et al.[14] in which information was gathered via email.
- Weaknesses
  Much of the data was gathered from patients’ recollection of past events and the information is therefore subject to recall bias. However, the gynaecologic symptoms (menorrhagia, dysmenorrhea and dyspareunia) persisted at the time of the consultation and could be evaluated more accurately.

## Conclusion

Women affected by hEDS present significant gynaecologic symptoms that are often are disabling, such as menorrhagia, dysmenorrhea, deep and intromission dyspareunia. Endometriosis may be incorrectly diagnosed on the basis of these symptoms, thereby leading to inappropriate treatment. The obstetric outcomes were mostly reassuring in this population. Furthermore, hormones may play a modulatory effect in this syndrome, and their influence merits further study.

## Abbreviations

CHC: Combined hormonal contraception
EDS: Ehlers Danlos syndrome
hEDS: Hypermobile type Ehlers Danlos syndrome
JHS: Joint hypermobile syndrome
MHT: Menopausal hormone therapy
MRI: Magnetic resonance imaging
POP: Progestin-only contraceptives

## References

[1] Beighton P, De Paepe A, Steinmann B, Tsipouras P, Wenstrup RJ (1998). Ehlers-Danlos syndromes: revised nosology, Villefranche, 1997. Ehlers-Danlos National Foundation (USA) and Ehlers-Danlos Support Group (UK). Am J Med Genet.

[2] Tinkle BT, Bird HA, Grahame R, Lavallee M, Levy HP, Sillence D (2009). The lack of clinical distinction between the hypermobility type of Ehlers-Danlos syndrome and the joint hypermobility syndrome (a.k.a. hypermobility syndrome). Am J Med Genet A.

[3] Castori M, Morlino S, Ghibellini G, Celletti C, Camerota F, Grammatico P (2015). Connective tissue, Ehlers-Danlos syndrome(s), and head and cervical pain. Am J Med Genet C Semin Med Genet.

[4] Castori M, Sperduti I, Celletti C, Camerota F, Grammatico P (2011). Symptom and joint mobility progression in the joint hypermobility syndrome (Ehlers-Danlos syndrome, hypermobility type). Clin Exp Rheumatol.

[5] Hakim et grahame, Nosology and inheritance pattern(s) of joint hypermobility syndrome and Ehlers-Danlos syndrome, hypermobility type: a study of intrafamilial and interfamilial variability in 23 Italian pedigrees. Castori M, Dordoni C, Valiante M, Sperduti I, Ritelli M, Morlino S, Chiarelli N, Celletti C, Venturini M, Camerota F, Calzavara-Pinton P, Grammatico P, Colombi M. Am J Med Genet A. 2014 Dec;164A(12):3010–20. 10.1002/ajmg.a.36805 25338840

[6] Castori M, Dordoni C, Valiante M, Sperduti I, Ritelli M, Morlino S (2014). Nosology and inheritance pattern(s) of joint hypermobility syndrome and Ehlers-Danlos syndrome, hypermobility type: A study of intrafamilial and interfamilial variability in 23 Italian pedigrees. Am J Med Genet A.

[7] Hakim AJ, Sahota A (2006). Joint hypermobility and skin elasticity: the hereditary disorders of connective tissue. Clin Dermatol.

[8] Malfait F, Hakim AJ, De Paepe A, Grahame R (2006). The genetic basis of the joint hypermobility syndromes. Rheumatol Oxf Engl.

[9] Sorokin Y, Johnson MP, Rogowski N, Richardson DA, Evans MI (1994). Obstetric and gynecologic dysfunction in the Ehlers-Danlos syndrome. J Reprod Med.

[10] Castori M, Morlino S, Dordoni C, Celletti C, Camerota F, Ritelli M (2012). Gynecologic and obstetric implications of the joint hypermobility syndrome (a.k.a. Ehlers-Danlos syndrome hypermobility type) in 82 Italian patients. Am J Med Genet A.

[11] Malfait F, De Paepe A (2009). Bleeding in the heritable connective tissue disorders: mechanisms, diagnosis and treatment. Blood Rev.

[12] Jackson SC, Odiaman L, Card RT, van der Bom JG, Poon M-C (2013). Suspected collagen disorders in the bleeding disorder clinic: a case-control study. Haemoph Off J World Fed Hemoph.

[13] Mcintosh LJ, Mallett VT, Frahm JD, Richardson DA, Evans MI (1995). Gynecologic disorders in women with Ehlers-Danlos syndrome. J Soc Gynecol Investig.

[14] Hurst BS, Lange SS, Kullstam SM, Usadi RS, Matthews ML, Marshburn PB (2014). Obstetric and gynecologic challenges in women with Ehlers-Danlos syndrome. Obstet Gynecol.

[15] Heitz NA, Eisenman PA, Beck CL, Walker JA (1999). Hormonal changes throughout the menstrual cycle and increased anterior cruciate ligament laxity in females. J Athl Train.

[16] Zeitoun J-D, Lefèvre JH, de Parades V, Séjourné C, Sobhani I, Coffin B (2013). Functional digestive symptoms and quality of life in patients with Ehlers-Danlos syndromes: results of a national cohort study on 134 patients. PLoS One.

[17] Guilleminault C, Primeau M, Chiu H-Y, Yuen KM, Leger D, Metlaine A (2013). Sleep-disordered breathing in Ehlers-Danlos syndrome: a genetic model of OSA. Chest.

[18] Hamonet C, Frédy D, Lefèvre JH, Bourgeois-Gironde S, Zeitoun J-D (2016). Brain injury unmasking Ehlers-Danlos syndromes after trauma: the fiber print. Orphanet J Rare Dis.

[19] Sundell G, Milsom I, Andersch B (1990). Factors influencing the prevalence and severity of dysmenorrhoea in young women. Br J Obstet Gynaecol.

[20] Ju H, Jones M, Mishra G (2014). The prevalence and risk factors of dysmenorrhea. Epidemiol Rev.

[21] Janssen EB, Rijkers ACM, Hoppenbrouwers K, Meuleman C, D’Hooghe TM (2013). Prevalence of endometriosis diagnosed by laparoscopy in adolescents with dysmenorrhea or chronic pelvic pain: a systematic review. Hum Reprod Update.

[22] Indicateur conjoncturel de fécondité [Internet]. Ined - Institut national d’études démographiques. 2016 [cited 2016 Jan 6]. Available from: https://www.ined.fr/fr/tout-savoir-population/chiffres/europe-pays-developpes/indicateurs-fecondite/

[23] Berkane N, Fiori O, Uzan S. Bilan à réaliser devant des fausses couches à répétition du premier trimestre. In: mises à jour en gynécologie et obstétrique. 2006. p. 127–42. http://www.cngof.asso.fr/d_livres/2006_GO_127_berkane.pdf.

[24] De Wandele I, Calders P, Peersman W, Rimbaut S, De Backer T, Malfait F (2014). Autonomic symptom burden in the hypermobility type of Ehlers-Danlos syndrome: a comparative study with two other EDS types, fibromyalgia, and healthy controls. Semin Arthritis Rheum.

[25] Hansen M, Kongsgaard M, Holm L, Skovgaard D, Magnusson SP, Qvortrup K (2009). Effect of estrogen on tendon collagen synthesis, tendon structural characteristics, and biomechanical properties in postmenopausal women. J Appl Physiol Bethesda Md 1985.

[26] Park S-K, Stefanyshyn DJ, Loitz-Ramage B, Hart DA, Ronsky JL (2009). Changing hormone levels during the menstrual cycle affect knee laxity and stiffness in healthy female subjects. Am J Sports Med.

[27] HAS, http://www.has-sante.fr/portail/upload/docs/application/pdf/2010-10/mesure_de_la_longueur_du_col_de_luterus_par_echographie_endovaginale_-_document_davis.pdf.

